# Applying feature selection and machine learning techniques to estimate the biomass higher heating value

**DOI:** 10.1038/s41598-023-43496-x

**Published:** 2023-09-26

**Authors:** Seyyed Amirreza Abdollahi, Seyyed Faramarz Ranjbar, Dorsa Razeghi Jahromi

**Affiliations:** 1 Faculty of Mechanical Engineering, University of Tabriz, Tabriz, Iran; 2 Department of Mechanical Engineering, Sharif University of Technology, Tehran, Iran

**Keywords:** Chemical engineering, Environmental sciences, Biomaterials

## Abstract

The biomass higher heating value (HHV) is an important thermal property that determines the amount of recoverable energy from agriculture byproducts. Precise laboratory measurement or accurate prediction of the HHV is essential for designing biomass conversion equipment. The current study combines feature selection scenarios and machine learning tools to establish a general model for estimating biomass HHV. Multiple linear regression and Pearson’s correlation coefficients justified that volatile matter, nitrogen, and oxygen content of biomass samples have a slight effect on the HHV and it is better to ignore them during the HHV modeling. Then, the prediction performance of random forest, multilayer and cascade feedforward neural networks, group method of data handling, and least-squares support vector regressor are compared to determine the intelligent estimator with the highest accuracy toward biomass HHV prediction. The ranking test shows that the multilayer perceptron neural network better predicts the HHV of 532 biomass samples than the other intelligent models. This model presents the outstanding absolute average relative error of 2.75% and 3.12% and regression coefficients of 0.9500 and 0.9418 in the learning and testing stages. The model performance is also superior to a recurrent neural network which was recently developed in the literature using the same databank.

## Introduction

Global warming and climate change originating from greenhouse gases (GHGs) are the main challenges of humankind in the current century^1,2^. Although there are various types of GHGs such as methane (CH_4_), carbon dioxide (CO_2_), nitrous oxide, hydrofluorocarbons, and chlorofluorocarbons, CO_2_ is considered as most dangerous GHGs^3–5^. Accordingly, the current increasing rate of GHGs in the atmosphere can result in a 3–5 °C temperature rise at the end of this century^6^. This temperature rise can contribute to some catastrophic results including storms, flooding, sea levels rising, and changes in precipitation patterns^7–9^. On the grounds, carbon capture and storage are required to reduce 2 °C the atmospheric temperature by 2100, based on Paris Agreement^10,11^.

To this end, biomass^12^, solar radiation^13^, hydropower^14^, geothermal^15^, and tidal^16^ have been nominated as the most common renewable energies, among them, biomass recently received significant attention, globally, because of low-cost, plentiful sources, accessibility, and desirable efficiency^17,18^. It is worth noting, currently, biomass is among the well-known source of energy, which by employing some mechanical-chemical treatments, including combustion, gasification^19^, or pyrolysis is converted to energy^12^. Accordingly, recently numerous studies have been devoted to the different aspects and characteristics of biomass conversion for being a source of energy. In this way, Skodras et al.^20^ investigated the specifications of combustion and pyrolysis processes for derived biomass from solid wastes. In another study, Arvidsson et al.^21^ evaluated the thermodynamic parameters and process characterizations of biomass gasification-based syngas to develop an oxo synthesis plant. The sintering and slagging stipulations of various sources of biomass from different regions of Europe were studied by Rodríguez et al.^22^ to produce a highly-efficient biofuel. However, higher heating value (HHV) is one of the key factors in designing and operating biomass-fueled energy systems^23^. Accordingly, an adiabatic oxygen bomb calorimeter is employed to measure fuel HHV, experimentally, while this technique is time-consuming and costly^24^. On the other hand, the outcomes of ultimate and/or proximate analyses can also be applied to obtain correlations to predict HHV. Nevertheless, proximate methodology concerning efficiency and cost has already demonstrated high potential to estimate HHV^25^.

Recent fascinating advances in machine learning (ML) tools resulted in their applications in different academic and industrial areas, including nanotechnology^26^, solar energy utilization^27^, energy efficiency^28^, renewable energy forecasting^29^, biomass, biofuels, and environmental preservation^30^. On the grounds, different topologies of ML such as artificial neural networks (ANNs)^31^, adaptive neuro-fuzzy inference systems (ANFIS)^32^, the support vector regression (SVR), random forest (RF), and group method of data handling (GMDH) have been widely applied to the paradigm design, data mining, fault tracing, and algorithm detection. Some researchers also suggested a combination of evolutionary techniques and ML tools for estimating a target parameter^33^. Accordingly, recently, numerous studies have studied the potential of different ML approaches for biomass-to-energy applications.

In this way, Olatunji et al.^34^ applied a multilayer perceptron neural network (MLPNN) to extract a black-box correlation between municipal solid waste HHV and its explanatory variables, i.e., moisture content, carbon, nitrogen, hydrogen, sulfur, oxygen, and ash. Karimi et al.^35^ employed different artificial intelligence (AI) scenarios to specify the heat capacity of a broad range of biomass (block and powder forms) by considering the temperature, the effect of biomass source, and appearance shape. Tsekos et al.^36^ considered the ANN model to derive the key parameters of lignocellulosic biomass pyrolysis related to the compositional and reaction criteria. Also, Ahmed et al.^37^ analyzed the effect of the moisture content on the characterization of biomass using different ML approaches. The estimation of the higher heating value of biomass from proximate was addressed by Xing et al.^38^ using the ANNs. In another attempt, Dashti et al.^39^ evaluated the possibility of utilizing the combination of the genetic algorithm (GA) and ANN/ANFIS to predict the biomass HHV based on proximate analysis. The activation energy as one of the main other thermal characterizations of biomass was estimated using ANN by Çepelioğullar et al.^40^. They reported that the ANNs have an excellent capacity with acceptable accuracy for the prediction of activation of energy of various biomass sources^40^.

This study is the first attempt to systematically select those biomass features that mainly govern the biomass HHV. Two well-established feature selection techniques are applied to identify the most important compositional features of biomass samples. The selected features are then considered as independent variables to compute biomass HHV utilizing five different machine-learning tools. The sensitivity analysis is then employed to determine the highest accurate tool to simulate the considered task. The selected model performance is then validated by a model that was recently proposed in the literature. The present study not only sorts the biomass proximate and ultimate compositional analyses based on their importance on the HHV, but it also is the most comprehensive work that has already been done in this field. The number of experimental records as well as the involved machine learning tools make this study the most generalized work about biomass HHV modeling. Indeed, the main novelty of the current study and the research gap is as follows:

- Previous works have randomly used either proximate or ultimate analysis or their combination to estimate biomass HHV. This study selects the most important explanatory variables among proximate and ultimate analyses using the well-known feature selection methods. Indeed, combining feature selection scenarios and machine learning methods is the most important novelty of the current research.
- Previous studies often proposed an empirical correlation or checked a small number of intelligent techniques to estimate biomass HHV. However, the present study applied several machine learning methods and selected the best one through ranking analysis.
- The accuracy of the constructed approach in the present study is better than a model recently suggested in the literature.

## Collected data from the literature

An extensive experimental database is needed to develop a general data-driven model capable of predicting a desired target (here, HHV). This database is also necessary to evaluate the model performance by diverse statistical criteria. On this ground, a literature databank including 532 HHV records as a function of proximate (fixed carbon, volatile matter, and ash) and ultimate (hydrogen, carbon, nitrogen, sulfur, and oxygen) compositional analyses was prepared. The supplementary material reports the numerical value of these variables and the source of each data sample.

## Machine learning methods

This section describes the fundamental basis of the machine learning tools that are applied to compute biomass HHV.

### Artificial neural network

Designing a reliable, accurate, and robust approach to extract the relation between input and output variables is a tough, onerous, and time-consuming mission that requires a detailed conception of the process^41^. In this way, artificial neural networks (ANNs) are suggested for such systems relying on the biological nervous systems of the human brain for function extraction, fault detection, and data mining^42,43^. Accordingly, this technique recently received a remarkable interest in different areas, specifically in the branches where getting experimental data is arduous^44^. One of the main benefits of the ANNs is related to constructing a trustworthy model between independent and dependent factors without any relation. Hence, interconnected processing units are employed to build the ANNs paradigm based on external information sources^44^. The multilayer perceptron neural network is one of the most favorable approaches^45^. To construct an MLPNN topology three main layers are required input, hidden, and output ones, which the input layer receives the main information from an external source which after some data treatment, transfers the information to the hidden layer, which here, the major data analysis and mathematical processing is employed. The operation defined by Eq. (1) is done in the neuron body^46^:

1 $$Z\, = \,\sum {w\,x\, + \,b}$$

where *x* is the entry signal and *w* is the weight vector by considering a bias (*b*) to specify the neuron’s output. Further, it is also required to choose a proper activation function ($\Psi$) which linear (Eq. 2), radial basis (Eq. 3), logarithmic sigmoid (Eq. 4), and hyperbolic tangent sigmoid (Eq. 5) are between the most popular ones^47^.

2 $$\Psi \left( Z \right) = \,Z$$

3 $$\Psi \left( Z \right) = \,\exp \left( { - 0.5\, \times \,Z^{2} /\,s^{2} } \right)$$

4 $$\Psi \left( Z \right) = \frac{1}{{1 + \exp \left( { - Z} \right)}}$$

5 $$\Psi \left( Z \right) = \frac{2}{{1 + \exp \left( { - 2\, \times \,Z} \right)}}\, - \,1$$

that $\Psi \left( Z \right)$ indicates the neuron’s output, *s* indicates the spread factor, and “*exp*” is the exponential function. It is worth noting that besides the MLPNN, the cascade feedforward neural network (CFFNN) is also one of the other well-known ANN types, which truly is a modified version of MLPNN that designs a network considering a direct connection among the input and output layers as well as concerning a non-straight connection with hidden layer^9,18^.

### Group method of data handling

The GMDH approach is a machine learning approach that provides the possibility to recognize data interrelations and effectively engineer the network configuration^48^. Accordingly, this topology has a robust potential to overcome the complexity of modeling in the processes with multi-inputs and single-output. To develop a GMDH model the defined neurons are related using a quadratic polynomial where the new neurons are generated in the next layer^49^. Routinely, the GMDH network connects the input and output layers through Volterra functional, series formula described by the Kolmogorov-Gabor polynomial, i.e., Eq. (6)^50^:

6 $$y^{cal} = \,a_{0} + \sum\limits_{i = 1}^{M} {a_{i} x_{i} } + \sum\limits_{i = 1}^{M} {\sum\limits_{z = 1}^{M} {a_{iz} x_{iz} } } + \sum\limits_{i = 1}^{M} {\sum\limits_{z = 1}^{M} {\sum\limits_{k = 1}^{M} {a_{izk} x_{izk} } } } + .....$$

here, *M* indicates the number of inputs, *x* is the input variables, and “*a*” is the coefficient. Afterward, the GMDH approach must be trained to minimize the square error (*SE*) between the real output (*y*) and the calculated output (*y*^*cal*^) according to Eq. (7)^50^:

7 $$SE\, = \,\sum\limits_{j = 1}^{N} {\left[ {y_{j}^{cal} - y_{j} } \right]}^{2}$$

The GMDH can ignore the combination of those coupled signals that introduce a relatively high uncertainty to predict the target variable.

### Random forest

The RF is one of the classifiers, which is constructed considering a group of decision trees known as weak learners that are required to be trained, parallelly, that can estimate the output concerning a majority-voting system^51^. In the RF, each decision tree strongly relies on a training dataset that is influenced by residual variation, noise, and particularity as uncertainties of data^52^. Accordingly, a minor variation in the training procedure has a significant effect on the development decision tree. However, an ensemble is employed to reduce the obstacles related to the decision tree algorithm. On the grounds, this strategy improves the accuracy of RF in comparison with a single decision tree as well as generalizes the potential of the developed approach, strongly^53^. However, to construct a more robust RF network employing heterogeneous decision trees with diversity accompanied by data particularity is required to be considered.

The required steps to design an RF paradigm are as follows^54^:

1. Step 1: The RF topology is developed with different sampling methods and considering the bootstrapping for employed replacement. On the other hand, it is necessary to generate n training sets after getting the experienced sample n times with n times.
2. Step 2: The element dataset is utilized to build n decision tree according to the obtained n training sets from Step 1.
3. Step 3: The single decision tree describes the features, and the best one is chosen by considering the Gini index, information divergence, and the ratio of divergence.
4. Step 4: Then the Random Forest is constructed based on the trained decision trees by considering the classification and regression analysis.

### Least-squares support vector regressor

The SVR is one of the other well-known ML approaches, which has a main feature than the common ANNs that minimizes the error using the higher bound extension, while in the other ones, the local error is considered^55^. Generally, the SVR analyzes the data using a large-scale quadratic relying on a linear decision surface assessment. Thus, to obviate the complexity of SVR, least-square SVR (LS-SVR) was developed and in this case, the optimization procedure is achieved using some linear equations instead of quadratic assessment^33^. In this way, the LS-SVR function is characterized by Eq. (8)^56^:

8 $$f(x) = \left\langle {\omega ,\phi (x)} \right\rangle + \,B$$

that, $\phi (x)$ indicates the kernel function, *ω* and *B* are the weight and bias of the model, respectively. On the hand, an optimization process is required for the cost function (Eqs. 9 and 10)^57^, as:

9 $$\min J\left( {\omega ,e} \right) = \frac{1}{2}\left\| \omega \right\|^{2} + \frac{1}{2}\gamma \sum\limits_{j = 1}^{N} {e_{j}^{2} }$$

10 $$s.t.\,\,\,\,\,\,\,y_{k} = e_{j} + < \omega ,\phi \left( {x_{j} } \right) > + \,B\,\,\,\,\,\,j = 1,......,N$$

Further, to assess the developed optimization the Lagrange function is employed (Eq. 11)^56^.

11 $$L_{LS - SVR} = \frac{1}{2}\left\| \omega \right\|^{2} + \frac{1}{2}\gamma \sum\limits_{j = 1}^{N} {e_{j}^{2} } - \sum\limits_{j = 1}^{N} {\alpha_{j}^{{}} } \left\{ {e_{j} + < \omega ,\phi \left( {x_{j} } \right) > + \,B\, - y_{j} } \right\}$$

To get the LS-SVR network, it is also required to solve Eq. (12)^57^:

12 $$\left\{ \begin{gathered} \omega = \sum\limits_{j = 1}^{N} {a_{j} } \phi \left( x \right) \hfill \\ \sum\limits_{j = 1}^{N} {a_{j} = 0} \hfill \\ a_{j} = \gamma e_{j} \,\,\,\,\,j = 1,2,....,N \hfill \\ y_{j} = \omega^{T} \phi \left( {x_{j} } \right) + B + e_{j} \,\,\,\,\,\,\,\,j = 1,2,....,N \hfill \\ \end{gathered} \right.$$

It is noteworthy that the established approach is based on the kernel function, calculated by Eq. (13)^56^:

13 $$\Omega_{lj} = \Phi \left( {x_{j} } \right)\Phi \left( {x_{1} } \right) = K\left( {x_{j} ,x_{1} } \right)\,\,\,\,\,\,\,\,\,\,l,j = 1,......,N$$

Several kernel functions, including quadratic, cubic, polynomial, linear, and Gaussian are possible to incorporate in the LS-SVR body.

## Results and discussions

Feature selection, machine learning construction/comparison, the best model selection, and performance evaluation are the main parts of the current section.

### Feature selection

As mentioned earlier, the literature tried to correlate biomass HHV with the proximate and ultimate compositional analyses of bio-samples. The present study applies two well-known feature selection methods, i.e., multiple linear regression and Pearson correlation coefficient to sort fixed carbon, volatile matter, ash, carbon, nitrogen, oxygen, sulfur, and hydrogen content of biomass samples based on their effect on the observed HHV.

#### Multiple linear regression (MLR)

The MLR is likely the most well-known feature selection method which is often integrated with machine learning tools to efficiently handle an advanced regression task^58^. The MLR aims to extract a linear relationship between a target and its influential variables. The magnitude and sign of the coefficient of each independent variable in the MLR clarify the strength and direction of its influence on the target function.

For the sake of simplicity, some notations are assigned to the proximate and ultimate compositional analyses of biomass samples and their counterpart HHV. Table 1 introduces the symbols allocated to the involved target and influential variables in the current study.

**Table 1:** Assigned notations to define independent and dependent variables.

Proximate analysis			Ultimate analysis					HHV (KJ/g)
Fixed carbon (wt%)	Volatile matter (wt%)	Ash (wt%)	Carbon (wt%)	Hydrogen (wt%)	Oxygen (wt%)	Nitrogen (wt%)	Sulfur (wt%)	
x_1_	x_2_	x_3_	x_4_	x_5_	x_6_	x_7_	x_8_	y

It should also be noted that the HHV and its influential variables have different magnitudes. Hence, it is necessary to normalize them before establishing the MLR. This normalization stage helps deduce the strength of the HHV relationship with independent variables solely based on their MLR coefficients. This study uses Eq. (14) to scale all biomass compositional characteristics into the same range of zero to + 1 ($\overline{x}$).

14 $$\overline{x}_{i,j} \, = \,\left( {x_{i,j} \, - \,x_{i}^{\min } } \right)/\left( {x_{i}^{\max } - \,x_{i}^{\min } } \right)\,\,\,\,\,\,\,and\,\,\,j = 1,2,\,...,\,N$$

where, *i* = 1, 2, 3, 4, 5, 6, 7, and 8 indicate fixed carbon, volatile matter, ash, carbon, hydrogen, oxygen, nitrogen, and sulfur content of biomaterials, respectively. In addition, *N* is the number of records. The superscripts *min* and *max* represent the minimum and maximum values of each variable.

The biomass HHV is also normalized into the [0 1] range applying Eq. (15). The normalized HHV is abbreviated by $\overline{y}$.

15 $$\overline{y}_{j} \, = \,\left( {y_{j} \, - \,y^{\min } } \right)/\left( {y^{\max } - \,y^{\min } } \right)\,\,\,\,\,\,\,j = 1,2,\,...,\,N$$

Equation (16) presents the mathematical expression of the MLR that linearly relates normalized HHV to its normalized influential variables.

16 $$\overline{{y_{j}^{cal} }} \, = \,\sum\nolimits_{i = 0}^{8} {A_{i} \, \times \,\overline{{x_{i,j} }} \,\,\,\,\,\,\,j = 1,2,...,N}$$

Table 2 introduces the coefficients of the constructed MLR. The negative values of A_3_, A_6_, and A_8_ clarify that the HHV decreases by the ash, oxygen, and sulfur content of biomass samples. On the other hand, fixed carbon, volatile matter, nitrogen, hydrogen, and carbon content of biomass samples result in increasing the HHV.

**Table 2:** Adjusted coefficients of the MLR equation.

A_0_	A_1_	A_2_	A_3_	A_4_	A_5_	A_6_	A_7_	A_8_
0.0324	0.2402	0.0667	− 0.3717	0.8606	0.2132	− 0.0616	0.0335	− 0.2036

The relative importance (RI) of the biomass compositional analysis can be easily computed using Eq. (17).

17 $$RI_{i} \, = \,abs\left( {A_{i} } \right)\, \times \,100/\sum\nolimits_{i = 1}^{8} {abs\left( {A_{i} } \right)}$$

**Figure 1:**
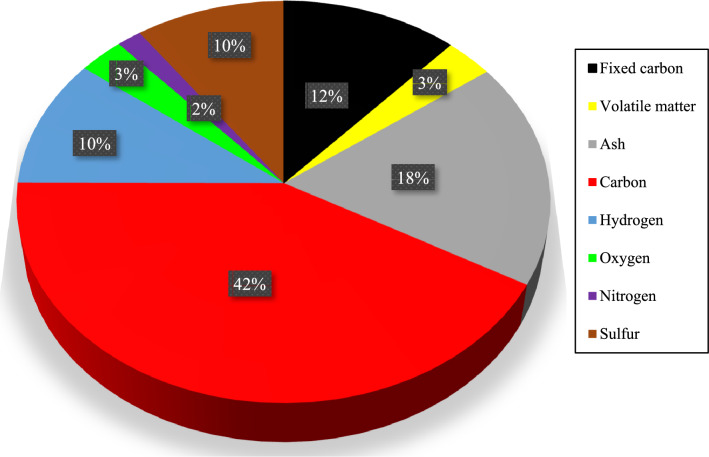
The relative importance of biomass compositions on the HHV.

The relative importance of each biomass ingredient on the observed HHV is illustrated in Fig. 1. This figure states that the nitrogen (2%), oxygen (3%), and volatile matter (3%) content of biomass samples have such a slight influence on the HHV that they can be ignored. This observation is due to the small coefficients of these biomass ingredients in the MLR, i.e., A_2_ = 0.0667, A_6_ =  − 0.0616, and A_7_ = 0.0335. Also, carbon (42%), ash (18%), fixed carbon (12%), hydrogen (10%), and sulfur (10%) content of biomass samples have a considerable effect on the HHV.

The MLR justified that it is better to model HHV solely based on the most important features, i.e., carbon, ash, fixed carbon, sulfur, and hydrogen content of biomass samples.

#### Pearson’s correlation coefficient

The Pearson correlation coefficient is another method that helps sort influential variables based on the importance of their relationship with a target function. Equation (18) introduces a mathematical way to calculate the Pearson coefficient ($\eta$) for a correlation between HHV and each influential variable.

18 $$\eta_{i} \, = \,\sum\nolimits_{j = 1}^{N} {\left( {x_{i,j} - x_{i}^{ave} } \right)\left( {y_{j} - \,y^{ave} } \right)} /\left( {\sqrt {\sum\nolimits_{j = 1}^{N} {\left( {x_{i,j} - \,x_{i}^{ave} } \right)^{2} } } \sqrt {\sum\nolimits_{j = 1}^{N} {\left( {y_{j} - \,y^{ave} } \right)^{2} } } } \right)$$

Here, *x*^*ave*^ and *y*^*ave*^ show the average value of influential and target variables, respectively. Equations (19) and (20) can be used to compute the average value of proximate/ultimate features and biomass HHV, respectively.

19 $$x_{i}^{ave} \, = \,\sum\nolimits_{j = 1}^{N} {x_{i,j} /N}$$

20 $$y^{ave} \, = \,\sum\nolimits_{j = 1}^{N} {y_{j} /N}$$

As Table 3 shows, Pearson’s coefficient for a correlation between a pair of variables ranges from − 1 to + 1. Similar to the MLR, the sign and magnitude of this coefficient clarify the direction and strength of the correlation, respectively.

**Table 3:** Pearson’s coefficients between each pair of involved variables in the present work.

	Fixed carbon	Volatile matter	Ash	Carbon	Hydrogen	Oxygen	Nitrogen	Sulfur	HHV
Fixed carbon	1.00								
Volatile matter	− 0.53	1.00							
Ash	− 0.12	− 0.70	1.00						
Carbon	0.35	0.13	− 0.51	1.00					
Hydrogen	− 0.10	0.41	− 0.48	0.18	1.00				
Oxygen	− 0.28	0.45	− 0.31	− 0.46	0.12	1.00			
Nitrogen	− 0.16	− 0.12	0.31	− 0.17	− 0.01	− 0.22	1.00		
Sulfur	− 0.07	− 0.18	0.31	− 0.24	− 0.14	0.04	0.12	1.00	
HHV	0.34	0.24	− 0.60	0.68	0.34	− 0.17	− 0.16	− 0.32	1.00

The last row of this table reports the HHV relationship with the composition of biomass ingredients. It can be seen that the biomass HHV has the weakest correlation with the nitrogen (− 0.16), oxygen (− 0.17), and volatile matter (0.24) content of biomass samples. These are exactly those variables that are identified by the MLR method as negligible features.

Furthermore, like the MLR method, Pearson’s method also identifies carbon, ash, fixed carbon, hydrogen, and sulfur content of biomass samples as the most important features. In summary, the feature selection accomplished by the MLR and Pearson’s methods clarifies that it is better to predict HHV solely as a function of carbon, ash, fixed carbon, hydrogen, and sulfur content of biomass samples and ignore all other ingredients of bio-samples.

### Designing the machine learning models

This section aims to design different machine learning tools (random forest, multilayer and cascade feedforward neural networks, group method of data handling, and least-squares support vector regressor) to predict biomass HHV based on those influential variables suggested by the feature selection methods. Then, the most accurate intelligent model is identified by comparing the performance of machine learning tools in the learning and testing stages.

All these machine learning tools have some coefficients that automatically adjust by an optimization algorithm. In addition, they have some hyperparameters that must be determined by trial-and-error procedure or other search techniques. Indeed, different machine learning models with different hyperparameters have been developed and their performances are monitored using statistical analyses. By comparing the achieved accuracy of models with different hyperparameters it is possible to determine the best hyperparameters. Interested readers may refer to Adedeji et al.^59^ study to find some techniques for hyperparameter tuning for machine learning models. Table 4 presents the most important hyperparameters of each machine-learning tool and the best ones selected through trial-and-error investigations.

**Table 4:** The summary of checked/selected hyperparameters of machine learning models.

Machine learning model	Checked hyperparameters	The best hyperparameter
MLPNN	Number of neuronic layers	2
	Number of neurons in each layer	13, 1
	Activation function in each layer	Tangent and logarithm sigmoid
	Optimization algorithm	Levenberg–Marquardt
CFFNN	Number of neuronic layers	2
	Number of neurons in each layer	14, 1
	Activation function in each layer	Logarithm and tangent sigmoid
	Optimization algorithm	Scaled Conjugate Gradient
LS-SVR	Kernel function kind	Gaussian
GMDH	Number of neuronic layers	3
	Number of neurons in each layer	7, 9, 1
RF	Number of trees in the forest	15
	Sampling method	Random with replacement
	Input method	Random input

This table indicates that the best MLPNN and CFFNN have two neuronic layers with the 5-13-1 and 5-14-1 configurations, respectively. The integer values in the MLPNN and CFFNN configurations show the number of influential variables, the number of hidden neurons, and the number of output neurons, correspondingly. These two ANNs include different activation functions in their neuronic layers and are trained by different optimization algorithms.

The kernel type is the only hyperparameter of the LS-SVR that must be determined by the trial-and-error process. Various kernel types, including linear, quadratic, cubic, polynomial, and Gaussian are checked, and the last candidate is identified as the best one.

The number of neuronic layers and the number of nodes in each layer are those GMDH hyperparameters that must be determined appropriately. The sensitivity analysis confirms that the GMDH with three neuronic layers and 5-7-9-1 configuration is superior to the other tested ones.

Finally, the trial-and-error analysis approves that 15 trees must be placed in the forest of the RF approach.

It should be mentioned that the following statistical criteria (Eqs. 21–24)^60^ are used to monitor the deviation between actual and predicted HHVs and determine the best hyperparameters of each machine-learning tool.

21 $$AARE\% \, = \,\left( {100/N} \right)\, \times \,\sum\nolimits_{j = 1}^{N} {abs\left( {y_{j} \, - \,y_{j}^{cal} } \right)/} y_{j}$$

22 $$MSE\, = \,\sum\nolimits_{j = 1}^{N} {\left( {y_{j} \, - \,y_{j}^{cal} } \right)^{2} /N}$$

23 $$RMSE\, = \,\left\{ {\sum\nolimits_{j = 1}^{N} {\left( {y_{j} \, - \,y_{j}^{cal} } \right)^{2} /N} } \right\}^{0.5}$$

24 $$R\, = \,\left\{ {1\, - \,\left( {\sum\nolimits_{j = 1}^{N} {\left( {y_{j} \, - y_{j}^{cal} } \right)^{2} /\sum\nolimits_{j = 1}^{N} {\left( {y_{j} \, - \,y^{ave} } \right)^{2} } } } \right)} \right\}^{0.5}$$

*AARE%*, *MSE*, *RMSE*, and *R* abbreviate absolute average relative error, mean squared error, root mean squared error, and regression coefficient, respectively. Furthermore, the *y*^*cal*^ superscript designates the calculated HHV.

To distinguish the machine learning tool with the highest accuracy toward HHV prediction, it is necessary to compare the performance of the selected models in the learning and testing stages. The 532 available datasets are randomly split into learning and testing categories with a ratio of 85/15. Indeed, the learning step of all the machine learning tools is accomplished by 452 datasets and the remaining 80 unseen samples are used to test the generalization capability of the trained models.

Table 5 summarizes the RF, LS-SVR, MLPNN, CFFNN, and GMDH performance for estimating the HHV records in the learning and testing steps. The AARE%, MSE, RMSE, and R criteria are used to monitor the model’s performance. Due to the availability of four statistical indexes and two different categories, it is not easy to identify the best model. Therefore, the next section uses the ranking test to sort the machine learning models based on their performance in the learning and testing phases.

**Table 5:** Performance of different machine learning models to predict learning/testing HHV data.

Machine learning model	Category	AARE%	MSE	RMSE	R
RF	Learning	3.88	1.27	1.13	0.8108
	Testing	3.88	1.27	1.13	0.8108
LS-SVR	Learning	3.49	0.83	0.91	0.8926
	Testing	4.26	1.02	1.01	0.8051
MLPNN	Learning	2.75	0.59	0.77	0.9500
	Testing	3.12	0.85	0.92	0.9418
CFFNN	Learning	2.73	0.54	0.73	0.9306
	Testing	4.62	1.36	1.17	0.7755
GMDH	Learning	4.37	1.31	1.14	0.8109
	Testing	4.58	1.48	1.22	0.8145

### Selecting the highest accurate machine learning model

The ranking test assigns the first rank (i.e., 1) to a model with the best observed statistical criterium (minimum value of AARE%, MSE, and RMSE, and maximum value of R). On the other hand, a model with the worst statistical criterium receives the last rank (i.e., 5). The second, third, and fourth ranks are also chronologically devoted to the other machine learning models. Then, it is possible to compute the average rank of a machine learning model from its ranks for the involved statistical indexes. Finally, the machine learning models are sorted based on their average performance in the learning and testing stages.

**Figure 2:**
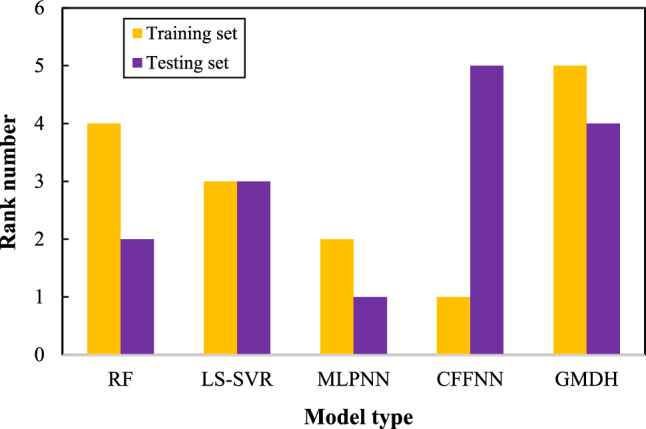
Ranking test to sort machine learning models based on their performance in the learning/testing stage.

Figure 2 presents the learning/testing rank of the investigated machine learning tools graphically. Although the CFFNN has the first rank in the learning stage (the best performance), it predicts the testing category so inaccurately that it places in the fifth rank position (the worst performance). Therefore, it is not feasible to consider the CFFNN the best model. The MLPNN with the second and first ranks in the learning and testing stages presents the best performance for estimating the biomass HHV. Also, the GMDH with the fifth and fourth ranks achieved in the learning and testing phases is the worst intelligent tool to predict the biomass HHV.

The performed ranking test approved that the MLPNN with a 5-13-1 configuration better predicts the biomass HHV than the other checked machine learning tools. The compatibility of actual HHVs and MLPNN predictions is approved by the excellent AARE = 2.75%, MSE = 0.59, RMSE = 0.77, and R = 0.9500 in the learning stage and AARE = 3.12%, MSE = 0.85, RMSE = 0.92, and R = 0.9418 in the testing step.

The subsequent sections comprehensively evaluate the MLPNN performance utilizing graphical and numerical analyses. In addition, the MLPNN accuracy will be compared with another model recently proposed in the literature^61^.

### Performance analysis

**Figure 3:**
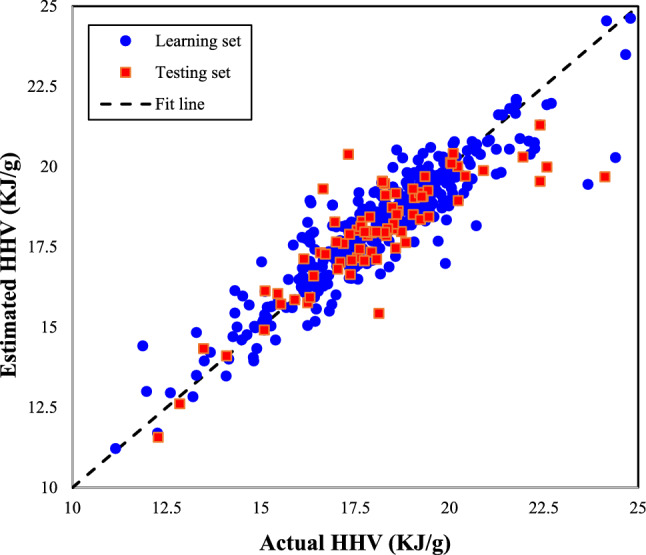
Correlation between actual and predicted HHVs of different biomass samples.

The scatter plot of computed biomass HHVs by the MLPNN versus their associated actual measurements for the learning and testing steps has been separately displayed in Fig. 3. This analysis approves excellent compatibility between the actual and computed target function. The regression coefficients of 0.9500 and 0.9418 observed in the learning and testing steps are also an indicator of the outstanding performance of the MLPNN to simulate the HHV of biomass samples with diverse origins.

**Figure 4:**
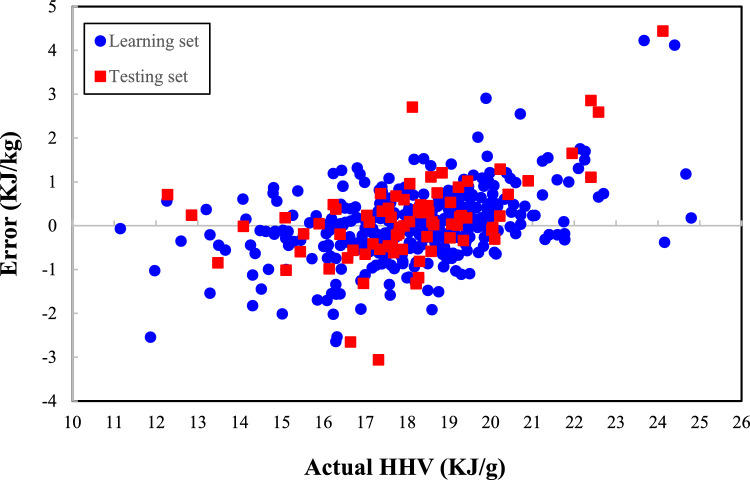
Performance checking of the MLPNN model in the learning and testing steps.

The performance of the suggested model for predicting the learning and testing sets has been monitored using the observed error between actual and computed biomass HHVs (Eq. 25) and the results are shown in Fig. 4.

25 $$e_{j} \, = \,y_{j} \, - y_{j}^{cal} \,\,\,\,\,\,\,j = 1,2,\,...,\,N$$

where *e* is an error. This investigation justifies that the observed errors between actual and predicted biomass HHVs are mainly between − 3 and 3 kJ/g. Furthermore, less than 1.2% of the actual HHV measurements have an absolute error of higher than 3 kJ/g.

Table 6 reports the main statistical characteristics (minimum, maximum, average, and standard deviation) of the error observed between actual and calculated biomass HHV. The MLPNN’s error for the biomass HHV estimation ranges from − 3.061 to 4.438 kJ/g.

**Table 6:** Summary of the MLPNN’s errors to predict the HHV records.

Variable	Minimum	Maximum	Average	SD
Error (KJ/g)	− 3.061	4.438	0.021	0.820

Moreover, the average and standard deviation (*SD*) of the observed errors is 0.021 and 0.820 kJ/g, respectively. Equations (26) and (27) define the SD and average (*e*^*ave*^) of the provided errors by the MLPNN.

26 $$e^{ave} \,\, = \,\sum\nolimits_{j = 1}^{N} {e_{j} } /N$$

27 $$SD\, = \,\left( {\sum\nolimits_{j = 1}^{N} {\left( {e_{j} \, - \,e^{ave} } \right)^{2} } /N} \right)^{0.5}$$

The previous visual and numerical investigations clarified that the MLPNN is a trustful tool to compute the HHV of bio-samples with a broad range of compositions.

### Validation by the literature model

The literature recently applied recurrent neural networks (RNN) to predict biomass HHV from all proximate and ultimate compositional analyses^61^. Therefore, it is a good idea to compare the prediction accuracy of this RNN with the proposed MLPNN in the current study. Table 7 compares the RNN and MLPNN performance to compute the learning/testing biomass HHVs utilizing AARE%, MSE, RMSE, and R indexes. It is easy to conclude that the MLPNN is more accurate than the recently constructed RNN in the literature.

**Table 7:** Comparing the MLPNN accuracy with the literature model.

Machine learning model	Group	AARE%	MSE	RMSE	R
MLPNN	Learning	2.75	0.59	0.77	0.9500
	Testing	3.12	0.85	0.92	0.9418
RNN	Learning	3.58	0.94	0.97	0.8834
	Testing	3.94	1.03	1.01	0.8226

**Figure 5:**
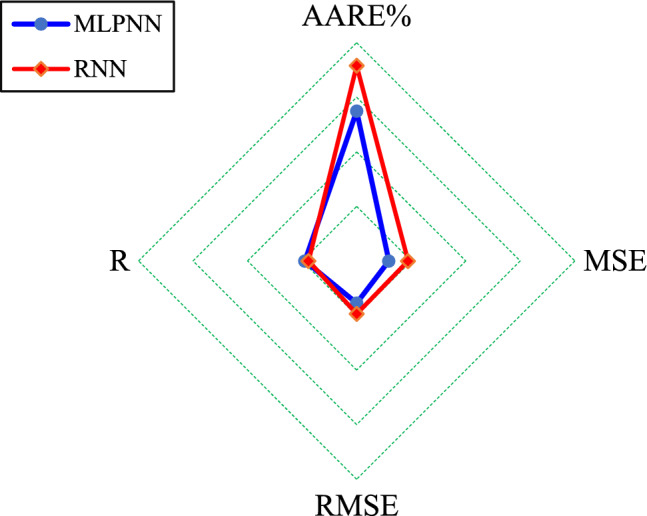
Comparing the MLPNN and RNN performance in the learning stage by Radar graph.

Now, the Radar graph is employed to visually compare the MLPNN and RNN performance in the learning and testing steps, respectively. Figure 5 shows that the obtained accuracies in terms of AARE%, MSE, RMSE, and R indices by the MLPNN are better than those provided by the RNN. It is better to highlight that small values of the first three indices and the R index close to unity are desirable from the modeling perspective.

**Figure 6:**
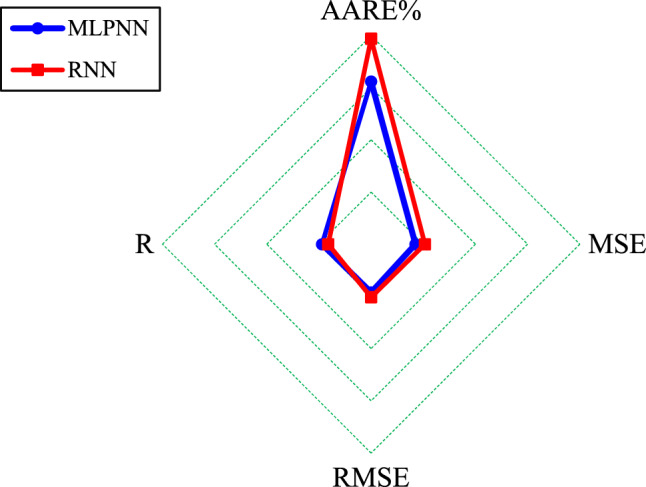
The Radar graph for comparing the MLPNN and RNN performance in the testing stage.

In addition, Fig. 6 displays that the MLPNN performance in terms of all four statistical indexes is superior to those obtained by the RNN during the testing stage.

## Conclusions

The literature has used a random combination of proximate and ultimate analyses to estimate the biomass HHV. Since the appropriate selection of the explanatory variables has a direct impact on the modeling accuracy, this work applied feature selection scenarios and machine learning methodologies to suggest a practical route to accurately predict the higher heating value of biomass samples. A relatively extensive experimental databank including 532 HHV records is used to validate the proposed method in the present study. The main findings of this research work can be summarized as follows:

- Multiple linear regression and Pearson’s correlation coefficient were applied to identify the most important influencing variables on the biomass HHV.
- Carbon and ash content are the main biomass ingredients to determine the HHV.
- HHV sharply increases by the carbon content and dramatically decreases by the ash content of biomass samples.
- Volatile matter and nitrogen/oxygen content of the biomass have a negligible effect on the HHV.
- Multilayer perceptron neural network provided more accurate prediction for the biomass HHV than the other five checked machine learning models.
- The MLPNN predicted 452 learning HHVs with the AARE = 2.75%, MSE = 0.59, RMSE = 0.77, and R = 0.9500.
- The model accuracy for predicting 80 unseen testing HHVs also approved by the AARE = 3.12%, MSE = 0.85, RMSE = 0.92, and R = 0.9418.
- The MLPNN provides more accurate HHV predictions than those obtained by RNN suggested in the literature.

### Supplementary Information

Supplementary Information.

## Data Availability

All the literature datasets analyzed in this study are available in the supplementary material.
